# Treatment of rubber wastewater using zinc sulphate as coagulants-data collection on removal efficiency for physicochemical and heavy metal parameters

**DOI:** 10.1016/j.dib.2020.106685

**Published:** 2020-12-22

**Authors:** Abbas F.M. Alkarkhi, Salem S. Abu Amr, Wasin A.A. Alqaraghuli, Yahya Özdemir, Muzafar Zulkifli, M.N. Mahmud

**Affiliations:** a Universiti Kuala Lumpur Business School (UniKL Bis), 50250, Kuala Lumpur, Malaysia; b Faculty of Engineering, Department of Environmental Engineering, Karabuk University, 78050, Karabuk, Turkey; c Skill Education center, PA, A-07-03 Pearl Avenue, Sungai Chua, 43000 Kajang, Selangor, Malaysia.; d Yalova University Safran Campus, Yalova Vocational School, Mehmet Durham street, 77200, No: 87, Center, Yalova, Turkey; e Green Chemistry and Sustainability Cluster, University Kuala Lumpur, Branch Campus Malaysian Institute of Chemical and Bioengineering Technology, Taboh Naning, 78000 Alor Gajah, Melaka, Malaysia.

**Keywords:** Wastewater, Zinc sulphate, Coagulant, Optimization, Response surface methodology, Analysis of variance (ANOVA)

## Abstract

This article provides data regarding the performance of zinc sulphate as a coagulant for treating rubber industry wastewater. The effect of four factors on removal efficiency of nine parameters is investigated, namely: pH, mixing speed, dosage of coagulant (zinc sulphate) and retention time. Response surface methodology was used to investigate the effect of selected variables. The data obtained from face centered composite design (FCCD) were analyzed by using analysis of variance (ANOVA) and regression model to find the optimum operating conditions for the selected factors.

## Specifications Table

**Table:** 

Subject area	Environmental engineering
More specific	Industrial wastewater treatment
Subject area	
Type of data	Table and figure
How data was acquired	Laboratory experiments and site sampling
Data format	Experimental data and analysis
Parameters for data collection	Wastewater sample collection, laboratory analysis, coagulant materials, coagulation using jar test
Description of data collection	Different dosages of zinc sulphate, pH, time and mixing speed, The physiochemical parameters are chemical oxygen demand (COD), total suspended solid (TSS), ammoniacal nitrogen (NH3-N), color and heavy metals (Pb, Fe, Zn, Cu, K)
Data Source location	University of Kuala Lumpur, Malaysian Institute of Chemical and Bio Engineering Technology (UniKL- MICET), Melaka, Malaysia. Gloves manufacturing company (Tan Sin Lian Industries Sdn. Bhd), Lot 179-184, Alor Gajah Industrial Estate (Phase III), Jalan Industri 7, Alor Gajah, 78000 Melaka, Malaysia. 2°21′43.5"N 102°12′17.6"E
Data accessibility	Within this article
Related research article	Tawfiq J. H. Banch, Marlia M. Hanafiah, Abbas F. M. Alkarkhi, Salem S. A. Amr, Nurul U. M. Nizam, (2020). Evaluation of Different Treatment Processes for Landfill Leachate Using Low‐Cost Agro‐Industrial Materials. Processes, 8, 111; 1-12, [1]. https://doi.org/10.3390/pr8010111

## Value of the Data

• The data produced an efficient method for rubber wastewater treatment using zinc sulphate as a coagulant.
• The data has benefits for rubber industries to manage their wastewater effluents. Also the data provides significant knowledge and applications to the university postgraduate students and research centres.
• The data provides model that can be used for treatment of several types of industrial wastewater treatment.

## 1 Data Description

**Fig. 1:**
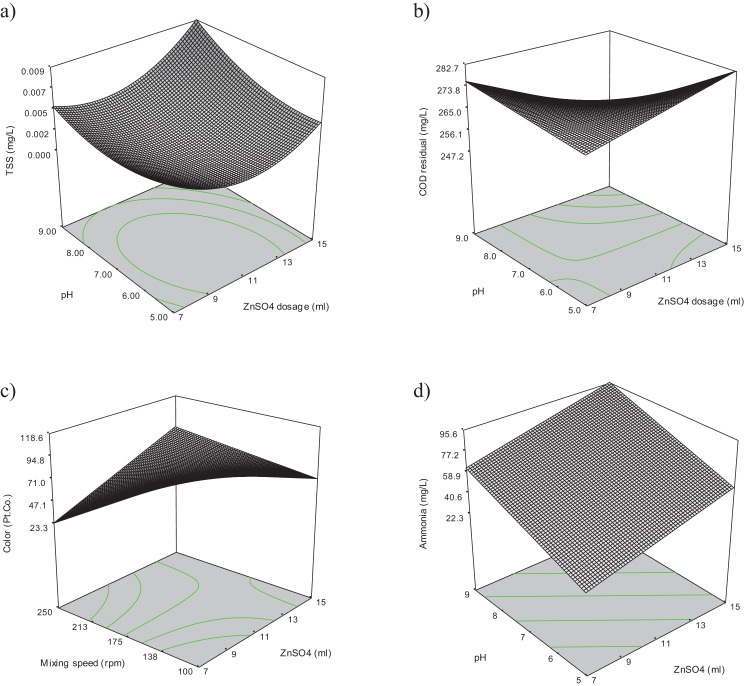
Response surface curves for the effect of two factor interaction on a)TSS, B) COD, C) Color and D) Ammonia.

**Fig. 2:**
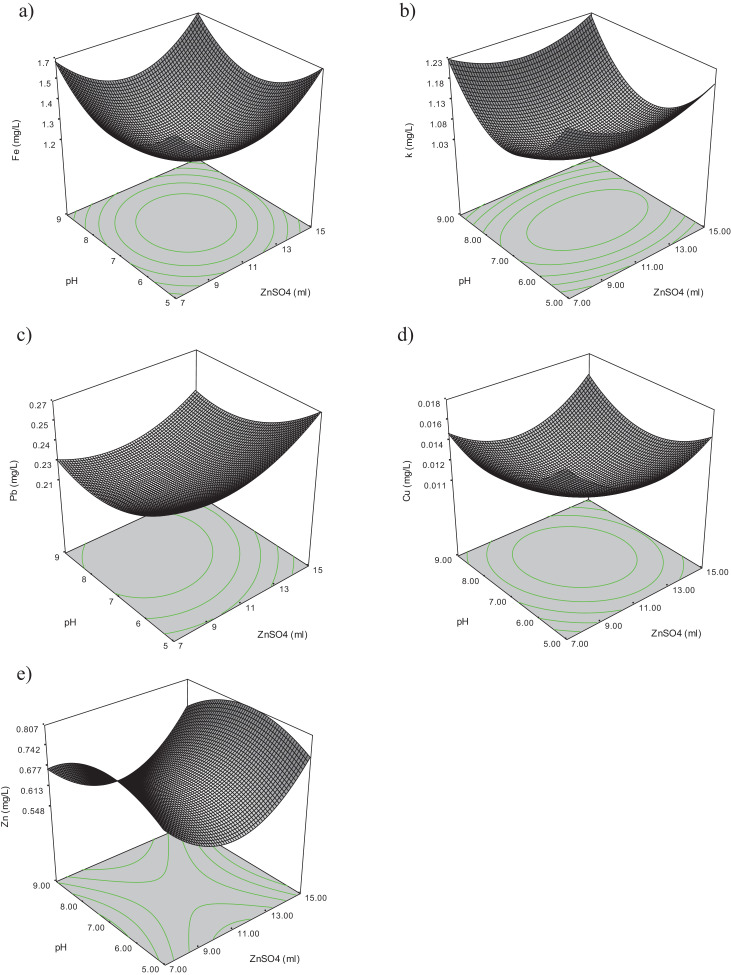
Response surface curves for the effect of two factor on a)Fe, B) K, C) Pb, D) Cu and E) Zn.

**Fig. 3:**
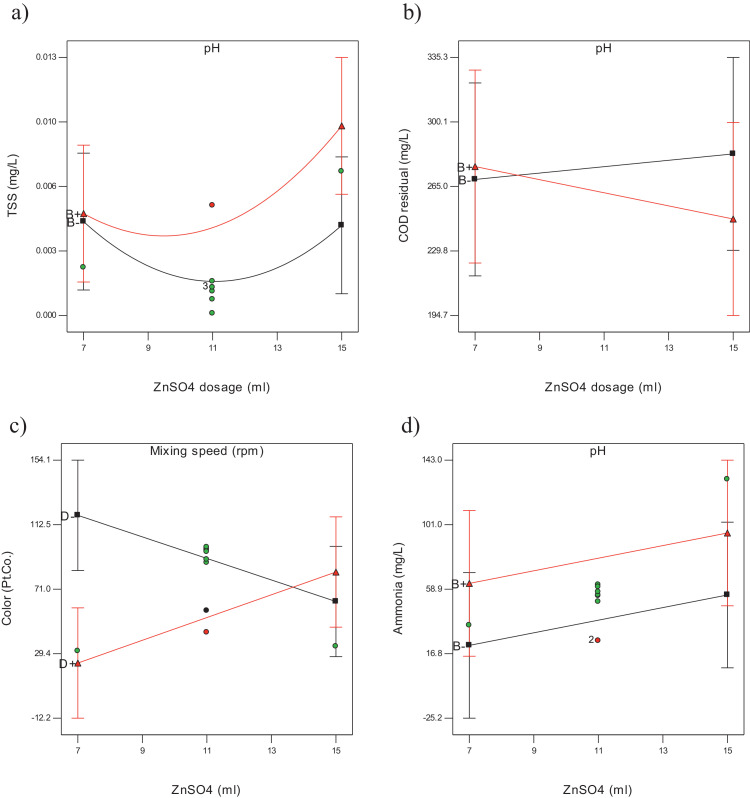
Interaction curves showing the behavior of two factors on the effect of a)TSS, b) COD, c) Color and d) Ammonia.

**Fig. 4:**
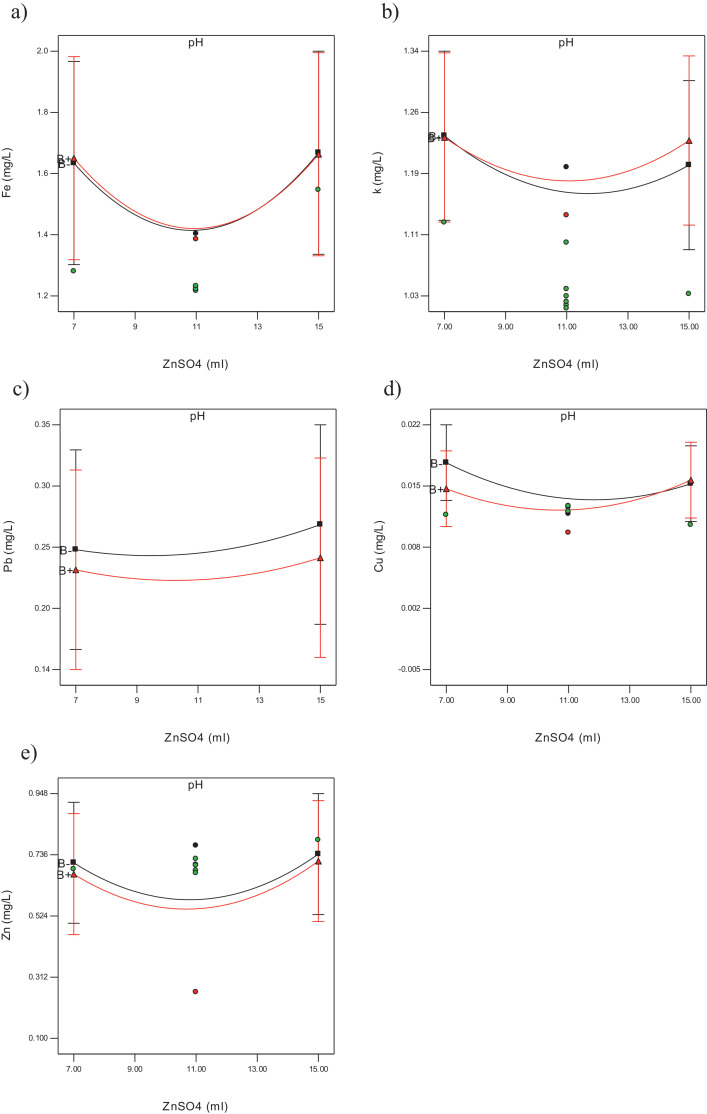
Interaction curves showing the behavior of two factors on the effect of a) Fe, b) K, c) Pb, d) Cu and e) Zn.

The raw data for thirty experiments using face centered composite design (FCCD), [2] covering all possible combinations of the selected variables (Dosage of zinc sulphate (A), pH (B), retention time (C) and mixing speed (D)) regarding measuring physiochemical parameters such as chemical oxygen demand (COD), total suspended solid (TSS), ammoniacal nitrogen (NH_3_-N), color and heavy metals (Pb, Fe, Zn, Cu, K) for rubber wastewater are presented in Table 1. The results of the experiments were analyzed using analysis of variance (ANOVA) [3]. The independent variables (factors) and corresponding levels used for optimization of rubber wastewater treatment is summarized in Table 2. The three-dimensional response surface curves and their effect on TSS, COD, Color, Ammonia heavy metals are presented in Fig. 1, Fig. 2. Interaction curves showing the behavior of two factors on the effect of TSS, COD, Color, Ammonia and heavy metals are presented in Fig. 3, Fig. 4. The significance of the influential variables is presented in Table 3 and 4 (analysis of variance (ANOVA)). Mathematical models that show the effect of significant variables on selected parameters are presented in Table 5 and 6 respectively. The equations of coded factors for TSS, COD, Color, ammonia and heavy metals removal were presented in Tables 5 and 6, respectively.

**Table 1:** The results of CCD including input variables and nine responses using zinc sulphate for treating rubber wastewater.

A = Dosage of zinc sulfate	B = pH	C = Retention time	D = Mixing speed	TSS	COD	Color	Ammonia	Fe	K	Pb	Cu	Zn
11	7	60	175	0.001	264	98	55	1.206	1.034	0.2401	0.013	7.001
7	9	90	250	0.002	143	27	27.8	−0.052	1.099	−0.046	0.001	0.624
11	7	60	250	0.001	228	43	15.8	1.324	1.261	0.2907	0.013	7.394
7	9	90	100	0.006	369	69	112	0.013	0.9788	0.032	0.001	1.079
15	5	90	100	0.005	169	68	50.4	0.01	0.9632	0.069	0.001	2.755
15	9	90	100	0.006	186	46	30.24	0.011	0.9359	0.05	0.002	1.331
11	7	30	175	0	252	92	9.52	0.013	0.9012	0.012	−0.005	2.494
7	5	30	250	0.001	155	37	105.84	0.011	1.171	0.063	0.002	2.469
15	9	30	250	0.002	155	64	25.2	0.012	1.036	0.015	−0.002	1.069
11	9	60	175	0.005	228	30	25.2	1.378	1.135	0.2239	0.01	0.2597
15	5	30	100	0.001	634	142	3.36	−0.034	0.9831	0.034	0.004	2.551
11	7	60	175	0	255	90	60.3	1.212	1.027	0.2342	0.013	7.012
15	7	60	175	0.007	204	34	130.5	1.542	1.037	0.288	0.011	7.868
7	9	30	100	0.003	490	14	5.6	0.027	1.024	0.017	0.003	1
11	7	60	175	0.001	249	95	57.1	1.222	1.019	0.24	0.012	6.987
7	9	30	250	0	199	24	75.6	0.009	1.098	0.036	−0.001	2.163
7	7	60	175	0.002	206	31	35.28	1.271	1.126	0.1383	0.012	6.86
7	5	90	250	0.001	213	8	90.72	−0.054	1.092	0.039	0.004	2.369
15	9	90	250	0.002	201	26	443.52	−0.057	1.239	−0.05	0.004	0.741
11	7	60	175	0.001	263	88	50.7	1.221	1.043	0.2319	0.013	7.213
7	5	30	100	0.001	632	354	19.04	−0.04	0.9499	−0.004	0.007	2.378
11	5	60	175	0.001	224	50	25.2	1.396	1.195	0.2028	0.012	7.676
15	5	30	250	0.001	180	186	55.44	0.009	1.07	0.07	−0.002	2.69
15	5	90	250	0.001	252	54	30.24	−0.051	1.043	−0.022	0.003	2.57
15	9	30	100	0.018	542	10	39.2	−0.04	1.008	−0.001	0.004	2.164
11	7	90	175	0	229	70	12.32	0.008	0.7462	0.027	−0.005	2.539
11	7	60	100	0.002	236	57	19.6	−0.019	0.8421	0.014	0.003	2.375
7	5	90	100	0.007	182	52	40.32	0.002	0.9582	0.033	0.002	2.46
11	7	60	175	0.001	256	96	61.7	1.211	1.101	0.2329	0.013	6.721
11	7	60	175	0.002	263	97	54.5	1.209	1.023	0.2331	0.013	6.81

**Table 2:** Independent variables (factors) and corresponding levels used for optimization.

		Range and levels		
Variables	Symbol	Low level	Center	High level
Coded		−1	0	1
Zinc Sulfate dosage	A	7 ml	11 ml	15 ml
pH	B	5	7	9
Reaction time	C	30	60	90
Mixing speed (rpm	D	100	175	250

**Table 3:** Analysis of variance for TSS, COE, Color and ammonia removal

**TSS**	**Sum of Mean F** **Source Squares DF Square Value Prob > F** Model 2.769E-004 14 1.978E-005 3.35 0.0133 *A 1.985E-005 1 1.985E-005 3.36 0.0868* *B 3.254E-005 1 3.254E-005 5.50 0.0331* *C 4.356E-007 1 4.356E-007 0.074 0.7897* *D 8.756E-005 1 8.756E-005 14.81 0.0016* *A2 2.176E-005 1 2.176E-005 3.68 0.0742* *B2 6.211E-006 1 6.211E-006 1.05 0.3216* *C2 7.951E-006 1 7.951E-006 1.35 0.2643* *D2 9.382E-007 1 9.382E-007 0.16 0.6959* *AB 2.093E-005 1 2.093E-005 3.54 0.0794* *AC 2.328E-005 1 2.328E-005 3.94 0.0658* *AD 6.631E-006 1 6.631E-006 1.12 0.3063* *BC 1.702E-005 1 1.702E-005 2.88 0.1104* *BD 1.743E-005 1 1.743E-005 2.95 0.1065* *CD 3.062E-008 1 3.062E-008 5.181E-003 0.9436* Residual 8.866E-005 15 5.911E-006 Total 3.656E-004 29

**COD**	**Sum of Mean F** **Source Squares DF Square Value Prob > F** Model 4.122E+005 10 41224.29 8.63 < 0.0001 *A 242.00 1 242.00 0.051 0.8243* *B 910.22 1 910.22 0.19 0.6673* *C 93168.06 1 93168.06 19.51 0.0003* *D 1.632E+005 1 1.632E+005 34.18 < 0.0001* *AB 1806.25 1 1806.25 0.38 0.5458* *AC 1122.25 1 1122.25 0.24 0.6334* *AD 3025.00 1 3025.00 0.63 0.4359* *BC 5550.25 1 5550.25 1.16 0.2945* *BD 324.00 1 324.00 0.068 0.7973* *CD 1.429E+005 1 1.429E+005 29.92 < 0.0001* Residual 90734.05 19 4775.48 Total 5.030E+005 29

**Color**	**Sum of Mean F** **Source Squares DF Square Value Prob > F** Model 86161.10 10 8616.11 3.94 0.0050 *A 10.89 1 10.89 4.979E-003 0.9445* *B 22826.72 1 22826.72 10.44 0.0044* *C 14056.06 1 14056.06 6.43 0.0202* *D 6536.06 1 6536.06 2.99 0.1001* *AB 10.56 1 10.56 4.830E-003 0.9453* *AC 264.06 1 264.06 0.12 0.7320* *AD 13053.06 1 13053.06 5.97 0.0245* *BC 21978.06 1 21978.06 10.05 0.0050* *BD 6930.56 1 6930.56 3.17 0.0910* *CD 495.06 1 495.06 0.23 0.6396* Residual 41548.77 19 2186.78 Total 1.277E+005 29

**NH_3_-N**	**Sum of Mean F** **Source Squares DF Square Value Prob > F** Model 42867.16 4 10716.79 1.92 0.1383 *A 4864.27 1 4864.27 0.87 0.3595* *B 7352.80 1 7352.80 1.32 0.2619* *C 13820.09 1 13820.09 2.48 0.1282* *D 16830.01 1 16830.01 3.02 0.0948* Residual 1.395E+005 25 5581.53 Total 1.824E+005 29

**Table 4:** Analysis of variance for heavy metals removal.

**Fe^+2^**	**Sum of Mean F** **Source Squares DF Square Value Prob > F** Model 11.08 14 0.79 13.73 < 0.0001 *A 2.568E-003 1 2.568E-003 0.045 0.8356* *B 1.502E-004 1 1.502E-004 2.607E-003 0.9600* *C 1.043E-003 1 1.043E-003 0.018 0.8948* *D 0.083 1 0.083 1.44 0.2492* *A2 0.15 1 0.15 2.61 0.1273* *B2 0.13 1 0.13 2.20 0.1587* *C2 3.46 1 3.46 60.01 < 0.0001* *D2 0.68 1 0.68 11.85 0.0036* *AB 4.622E-004 1 4.622E-004 8.022E-003 0.9298* *AC 2.560E-004 1 2.560E-004 4.443E-003 0.9477* *AD 1.822E-004 1 1.822E-004 3.163E-003 0.9559* *BC 1.822E-004 1 1.822E-004 3.163E-003 0.9559* *BD 3.610E-004 1 3.610E-004 6.265E-003 0.9380* *CD 8.930E-003 1 8.930E-003 0.15 0.6994* Residual 0.86 15 0.058 Total 11.94 29

**K**	**Sum of Mean F** **Source Squares DF Square Value Prob > F** Model 0.26 14 0.019 3.33 0.0136 *A 1.834E-003 1 1.834E-003 0.33 0.5759* *B 9.145E-004 1 9.145E-004 0.16 0.6921* *C 1.920E-003 1 1.920E-003 0.34 0.5672* *D 0.12 1 0.12 21.28 0.0003* *A2 7.063E-003 1 7.063E-003 1.26 0.2795* *B2 0.048 1 0.048 8.51 0.0106* *C2 0.11 1 0.11 19.52 0.0005* *D2 1.284E-003 1 1.284E-003 0.23 0.6392* *AB 1.071E-003 1 1.071E-003 0.19 0.6684* *AC 2.473E-003 1 2.473E-003 0.44 0.5168* *AD 1.645E-004 1 1.645E-004 0.029 0.8663* *BC 2.609E-003 1 2.609E-003 0.47 0.5057* *BD 8.556E-007 1 8.556E-007 1.525E-004 0.9903* *CD 3.218E-003 1 3.218E-003 0.57 0.4606* Residual 0.084 15 5.609E-003 Total 0.35 29

**Pb**	**Sum of Mean F** **Source Squares DF Square Value Prob > F** Model 0.29 14 0.021 5.57 0.0010 *A 1.163E-003 1 1.163E-003 0.31 0.5866* *B 2.401E-003 1 2.401E-003 0.64 0.4370* *C 6.722E-004 1 6.722E-004 0.18 0.6787* *D 1.278E-003 1 1.278E-003 0.34 0.5688* *A2 5.010E-004 1 5.010E-004 0.13 0.7204* *B2 5.155E-004 1 5.155E-004 0.14 0.7166* *C2 0.084 1 0.084 22.23 0.0003* *D2 5.697E-003 1 5.697E-003 1.51 0.2377* *AB 1.266E-004 1 1.266E-004 0.034 0.8570* *AC 1.806E-005 1 1.806E-005 4.796E-003 0.9457* *AD 1.463E-003 1 1.463E-003 0.39 0.5425* *BC 8.556E-005 1 8.556E-005 0.023 0.8822* *BD 1.620E-003 1 1.620E-003 0.43 0.5218* *CD 0.010 1 0.010 2.67 0.1232* Residual 0.056 15 3.766E-003 Total 0.35 29
**Cu**	**Sum of Mean F** **Source Squares DF Square Value Prob > F** Model 8.840E-004 14 6.314E-005 7.25 0.0002 *A 2.067E-006 1 2.067E-006 0.24 0.6332* *B 6.820E-006 1 6.820E-006 0.78 0.3902* *C 5.000E-007 1 5.000E-007 0.057 0.8139* *D 1.275E-006 1 1.275E-006 0.15 0.7074* *A2 2.026E-005 1 2.026E-005 2.33 0.1480* *B2 1.525E-005 1 1.525E-005 1.75 0.2055* *C2 4.802E-004 1 4.802E-004 55.13 < 0.0001* *D2 6.702E-007 1 6.702E-007 0.077 0.7853* *AB 1.056E-005 1 1.056E-005 1.21 0.2881* *AC 5.062E-006 1 5.062E-006 0.58 0.4576* *AD 6.250E-008 1 6.250E-008 7.176E-003 0.9336* *BC 1.563E-006 1 1.563E-006 0.18 0.6779* *BD 6.250E-008 1 6.250E-008 7.176E-003 0.9336* *CD 4.556E-005 1 4.556E-005 5.23 0.0371* Residual 1.306E-004 15 8.710E-006 Total 1.015E-003 29

**Zn**	**Sum of Mean F** **Source Squares DF Square Value Prob > F** Model 1.50 14 0.11 4.84 0.0022 *A 6.384E-003 1 6.384E-003 0.29 0.5993* *B 4.560E-003 1 4.560E-003 0.21 0.6566* *C 0.053 1 0.053 2.40 0.1425* *D 0.15 1 0.15 6.65 0.0210* *A2 0.053 1 0.053 2.39 0.1433* *B2 0.017 1 0.017 0.75 0.4010* *C2 0.30 1 0.30 13.67 0.0021* *D2 0.029 1 0.029 1.29 0.2734* *AB 2.265E-004 1 2.265E-004 0.010 0.9208* *AC 1.321E-003 1 1.321E-003 0.060 0.8104* *AD 1.201E-003 1 1.201E-003 0.054 0.8191* *BC 0.058 1 0.058 2.60 0.1278* *BD 0.081 1 0.081 3.64 0.0759* *CD 0.071 1 0.071 3.21 0.0934* Residual 0.33 15 0.022 Total 1.83 29

**Table 5:** Equations of coded factors for TSS, COD, Color and ammonia removal.

**TSS** = 1.501E-003 +1.050E-003 A +1.344E-003 B +1.556E-004 C -2.206E-003 D +2.898E-003 A2 +1.548E-003 B2 -1.752E-003 C2 -6.018E-004 D2 +1.144E-003 AB -1.206E-003 AC -6.437E-004 AD -1.031E-003 BC -1.044E-003 BD -4.375E-005 CD (3)

**COD** = +268.63 -3.67 A -7.11 B -71.94 C -95.22 D -10.63 AB -8.38 AC +13.75 A D +18.62 BC -4.50 BD +94.50 CD (4) **Color** = +71.73 +0.78 A -35.61 B -27.94 C -19.06 D +0.81 AB +4.06 AC +28.56 AD +37.06 B C +20.81 BD +5.56 CD (5)

**Ammonia** = +58.91 +16.44 A +20.21 B +27.71 C +30.58 D (6)

**Table 6:** Equations of coded factors for heavy metals removal.

**Fe** = +1.19 +0.012 A +2.889E-003 B -7.611E-003 C +0.068 D +0.24 A2+0.22 B2 1.16 C2 -0.51 D2 -5.375E-003 AB +4.000E-003 AC +3.375E-003 A D -3.375E-003 BC -4.750E-003 x B x D -0.024 x C x D (7)

**K** = +1.04 -0.010 A +7.128E-003 B -0.010 C +0.081 x D +0.052 A2 +0.14 B2 -0.21 C2 +0.022 D2 +8.181E-003 A B +0.012 AC -3.206E-003 AD +0.013 BC +2.313E-004 BD +0.014 CD (8)

**Pb** = +0.22 +8.039E-003 A -0.012 B -6.111E-003 C +8.428E-003 D +0.014 A2 +0.014 B2 -0.18 C2 -0.047 D2 -2.813E-003 AB -1.063E-003 AC -9.562E-003 AD -2.312E-003 BC -0.010 BD -0.025 CD (9)

**Cu** = +0.011 -3.389E-004 A -6.156E-004 B +1.667E-004 C -2.661E-004 D +2.796E-003 A2 +2.426E-003 B2 -0.014 C2 -5.086E-004 D2 +8.125E-004 AB +5.625E-004 A C -6.250E-005 AD +3.125E-004 BC -6.250E-005 B D +1.688E-003 CD (10)

**Zn** = +0.64 + 0.019 A -0.016 B +0.054 C +0.090 D +0.14 A2 -0.080 B2-0.34 C2 -0.11 x D2 +3.763E-003 AB +9.088E-003 AC -8.662E-003 AD +0.060 BC +0.071 BD +0.067 CD (11)

## 2 Experimental Design, Materials and Methods

### 2.1 Sampling

20 l sample of rubber wastewater was collected from Tan Sin Lian Industries Sdn. Bhd, one of the gloves manufacturing companies that located in Kawasan Perindustrian Melekek, Alor Gajah, Malaysia. This company is a global glove manufacturing that operates for the past ten years. The sample was collected directly from production factory during the period between April and June 2019. Then, the sample was stored in the sealed plastics bottles and preserved at a temperature less than 4 °C before being used and analyzed. Then the sample was characterized following standard methods for water and wastewater analysis [4].

### 2.2 Coagulation process by using ZnSO_4_

#### 2.2.1 Preparation of reagent

In this section rubber wastewater was coagulated using Zinc sulphate (ZnSO_4_). A set of ZnSO_4_ dosages were added to rubber wastewater samples gradually to determine the optimum conditions. The performance of the best dosage was selected based on COD, Color and NH_3_–N removal efficiencies. Orbital Shaker (Luckham R100/TW Rotatable Shaker 340 mm X 245 mm) with at 200 rpm was used for samples shaking [5]. All experiments were performed at room temperature (28 °C) using 100 mL of rubber wastewater samples placed in conical flasks with a 250 mL capacity. pH of the samples was controlled by using 3 M of sulphuric acid solution and sodium hydroxide solution, respectively [6]. All experiments were performed at laboratory of Malaysian Institute of chemical & Bioengineering Technology, University of Kuala Lumpur, Melaka, Malaysia.

### 2.3 Experimental design

Four factors, namely ZnSO_4_ dosage (A), pH (B), reaction time (C) and mixing ratio (D)are thought to be influential factors on nine responses TSS, COD, color, ammonia, Fe, K, Pb, Cu, and Zn, removal efficiency from rubber wastewater samples was tested and evaluated. Face centered composite design (FCCD) in response surface methodology (RSM) was used to investigate the effect of the four factors on the selected responses and find the optimum operating conditions for the four factors. The levels of selected factors were chosen based on literature and preliminary experiments, the actual and coded levels are given in Table 2.

The relationship between the selected factors (A,B,C, D) and each of the responses is usually described in response surface methodology (RSM) by a second-order polynomial as given in Eq. (1).

(1) $$Y=\beta_{0}+\sum_{i=1}^{4}\beta_{i}X_{i}+\sum_{i}^{4}\beta_{ii}X_{i}^{2}+\sum_{i<j}\beta_{ij}X_{ij}$$

where Y represents the dependent variable (TSS,COD,color,ammonia,Fe,K,Pb,Cu,Zn), $\beta_{0}$, $\beta_{i}$ and $\beta_{ii}$are linear coefficient, quadratic coefficient and interaction coefficients respectively, need to be estimated, and $X_{i}$ represents the independent variables (A,B,C,D).

All possible combination of selected factors (A, B, C, and D) to run FCCD is represented by thirty experiments distributed as follows: sixteen experiments for the factorial design, eight experiments are for axial (star) points and six experiments at the center of the design [2]. To avoid or minimize the effect of unexpected variability in the responses, the experiments were run in random order.

### 2.4 Analytical methods

COD, color and NH_3_–N, were immediately tested before and after each experiment using UV-VIS spectrophotometer (HACH DR 2800). Leachate sample was shacked well analyzed. NH_3_–N concentration was measured by the Phenol Method No. (4500) using a UV-VIS spectrophotometer at 640 nm with a light path of 1 cm or greater. pH was measured using a portable digital pH/Mv meter (Inolab pH 720, WTW 82362 Weilheim, Germany). COD concentration was determined by the open reflux method No. (5220). Heavy metals were tested by Atomic Absorption Spectroscopy (UNICAM 929 AA spectrometer). The test values are presented as the average of the three measurements, and the difference between the measurements of each value was less than 3%. The removal efficiencies of COD and NH3–N were obtained using the following Eq. (2):

(2) Removal(%)=[(Ci−−Cf)/Ci]×100

where Ci and Cf refer to the initial and final TSS, COD, Color and NH_3_–N concentrations, respectively.

## CRediT Author Statement

**Abbas F.M. Alkarkhi:** Writing, original draft preparation, Conceptualization, supervision; **Salem S. Abu Amr:** Writing, Data curation, Conceptualization, Methodology; **Wasin A.A. Alqaraghuli:** Writing, data curation, modeling; **Yahya Özdemir:** software, reviewing, editing; **Muzafar Zulkifli:** Writing, visualization, methodology; **M.N. Mahmud:** reviewing, editing .

## Declaration of Competing Interest

The authors declare that they have no known competing financial interests or personal relationships which have or could be perceived to have influenced the work reported in this article.

## References

[1] Banch T.J.H., Hanafiah M.M., Alkarkhi A.F.M., S.Abu Amr S., Nizam N.U.M. (2020). Evaluation of different treatment processes for landfill leachate using low-cost agro-industrial materials. Processes.

[2] Alkarkhi A.F.M., Alqaraghuli W.A.A., Yusup Y., Abu Amr S.S., Mahmud M.N., Dewayantoa Nugroho (2018). Data on the absorbance of glucose during the acid hydrolysis of the sugarcane bagasse. Data in Brief.

[3] Abu Amr S.S., Alkarkhi A.F.M., Yusup Y., Bashir M.J.K. (2019). Comparison and evaluation of different leachate treatment processes for chemical oxygen demand and color removals - statistical assessment. EnvironmentAsia.

[4] Standard Methods for the Examination of Water and Wastewater APHA, American Public Health Association (APHA), 21th ed., Washington, DC (2005).

[5] N.M.F.M. Yasin M.S., Hossain H.P.S., Abdul Khalil M., Zulkifli A., Al-Gheethi A.J., Asis A.N.A. (2020). Yahaya Treatment of palm oil refinery effluent using tannin as a polymeric coagulant: isotherm, kinetics, and thermodynamics analyses. Polymers.

[6] Onn S.W., Bashir M.J.K., Sethupathi Sumathi, Abu Amr S.S., Nguyen Tan Tai (2020). Colour and COD removal from mature landfill leachate using electro-persulphate oxidation process. Mater. Today.

